# Recognition‐Mediated Hydrogel Swelling Controlled by Interaction with a Negative Thermoresponsive LCST Polymer

**DOI:** 10.1002/anie.201605630

**Published:** 2016-10-12

**Authors:** Khaled Belal, François Stoffelbach, Joël Lyskawa, Matthieu Fumagalli, Dominique Hourdet, Alba Marcellan, Lieselot De Smet, Victor R. de la Rosa, Graeme Cooke, Richard Hoogenboom, Patrice Woisel

**Affiliations:** ^1^ Unité des Matériaux et Transformations, UMR CNRS 8207, ENSCLEquipe Ingénierie des Systèmes Polymères (ISP)59655Villeneuve O'Ascq CedexFrance; ^2^ Sorbonne Universités, UPMC Univ Paris 06, CNRSInstitut Parisien de Chimie MoléculaireUMR 8232, Equipe: chimie des polymères F-75252Paris Cedex 05France; ^3^ ESPCI ParisTech (PSL Research University) and UPMC Univ. Paris 06 (Sorbonne Universités)Sciences et Ingénierie de la Matière Molle (CNRS UMR 7615)10 Rue Vauquelin75005ParisFrance; ^4^ Supramolecular Chemistry GroupDepartment of Organic and Macromolecular ChemistryGhent UniversityKrijgslaan 281 S4-bis9000GhentBelgium; ^5^ WestCHEM, School of ChemistryUniversity of GlasgowGlasgowG12 8QQUK

**Keywords:** host–guest compounds, hydrogels, supramolecular chemistry, swelling, thermoresponsive polymers

## Abstract

Most polymeric thermoresponsive hydrogels contract upon heating beyond the lower critical solution temperature (LCST) of the polymers used. Herein, we report a supramolecular hydrogel system that shows the opposite temperature dependence. When the non‐thermosesponsive hydrogel NaphtGel, containing dialkoxynaphthalene guest molecules, becomes complexed with the tetra cationic macrocyclic host CBPQT^4+^, swelling occurred as a result of host–guest complex formation leading to charge repulsion between the host units, as well as an osmotic contribution of chloride counter‐ions embedded in the network. The immersion of NaphtGel in a solution of poly(N‐isopropylacrylamide) with tetrathiafulvalene (TTF) end groups complexed with CBPQT^4+^ induced positive thermoresponsive behaviour. The LCST‐induced dethreading of the polymer‐based pseudorotaxane upon heating led to transfer of the CBPQT^4+^ host and a concomitant swelling of NaphtGel. Subsequent cooling led to reformation of the TTF‐based host–guest complexes in solution and contraction of the hydrogel.

Controlled deformation processes are ubiquitous both in biological systems and materials science.^1^, ^2^ For example, in living systems, tissues or muscles may undergo expansion or contraction as a result of an injury or an external physiological stimulus.^3^ Similarly, synthetic smart polymer gels, capable of swelling or deswelling in response to various physical (e.g. temperature, light, electric and magnetic fields) and chemical stimuli (e.g. pH, ions, molecules),^4^, ^5^, ^6^ have been developed during the last decades.^7^, ^8^, ^9^, ^10^, ^11^, ^12^, ^13^, ^14^, ^15^, ^16^ Reversible supramolecular binding of guest molecules or metal ions has also been reported to promote isothermal polymer phase transitions leading to the contraction or expansion of hydrogels.^17^ Nevertheless, in the field of controlled responsive soft materials, temperature remains the most common trigger, and thermoresponsive hydrogels are attracting considerable attention for potential applications in artificial muscles, smart actuators, and drug‐delivery systems.^18^, ^19^, ^20^

Scientists frequently use the versatility of polymer chemistry to design macromolecular structures that exhibit temperature‐induced volume/phase transitions in aqueous environments. The polymer phase separation, which corresponds to the thermodynamic transition from a good solvent to a bad solvent, can be driven either by heating, for systems showing a lower critical solution temperature (LCST; negative thermoresponse), or by cooling, for those with an upper critical solution temperature (UCST; positive thermoresponse).^21^ The area of thermoresponsive hydrogels is largely dominated by LCST systems,^22^, ^23^, ^24^ whereby poly(*N*‐isopropylacrylamide) (PNIPAM)‐based hydrogels are the most common systems, as they undergo a sharp phase transition close to physiological temperatures (around 32 °C).^25^ In contrast, UCST‐type thermoresponsive hydrogels are much less developed,^26^, ^27^, ^28^, ^29^ even though hydrogen‐bonding UCST polymers seem very promising for biomedical applications.^30^

The goal of the present study was to develop a hydrogel system that undergoes abrupt swelling upon heating (positive thermoresponse) while remaining hydrophilic in both the contracted and expanded states. We designed a supramolecular hydrogel system featuring a non‐thermoresponsive poly(*N*,*N*‐dimethylacrylamide) hydrogel functionalized with dialkoxynaphthalene guest units (NaphtGel; Figure 1 a) that significantly swells upon complexation with the tetracationic cyclophane host cyclobis(paraquat‐*p*‐phenylene) (CBPQT^4+^). On the basis of our previous observation that the supramolecular association of CBPQT^4+^ and PNIPAM polymers containing electron‐rich guest units can be thermally controlled,^31^, ^32^, ^33^ we envisioned that the combination of NaphtGel with CBPQT^4+^ and a PNIPAM end‐functionalized with tetrathiafulvalene (TTF‐PNIPAM) as a competitive guest would lead to temperature‐induced swelling of NaphtGel (Figure 1 b). In this three‐component system, TTF‐PNIPAM will be complexed with CBPQT^4+^ below the LCST phase transition, as TTF is a more strongly binding guest than naphthalene. Heating of the system above the phase‐transition temperature of the TTF‐PNIPAM⋅CBPQT^4+^ complex will lead to the collapse of TTF‐PNIPAM and induce dethreading of the host–guest complex and release of the free cyclophane unit into the aqueous solution. The released CBPQT^4+^ will then diffuse towards the NaphtGel to form host–guest complexes with the naphthalene units, thus leading to the swelling of NaphtGel. An interesting aspect of this supramolecular gel system is that the temperature‐induced transfer of CBPQT^4+^ from TTF‐PNIPAM to NaphtGel (and vice versa) yields a distinct visual output signal in the form of a change in color, as the complexes of CBPQT^4+^ with naphthalene and TTF have very different colors.

**Figure 1:**
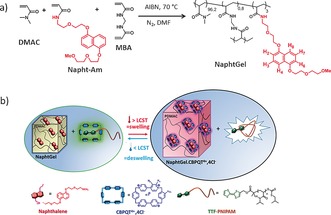
a) Synthesis of NaphtGel (note that after synthesis, the NaphtGel hydrogel was obtained by replacing the DMF with water). b) Illustration of the heating‐induced swelling of the three‐component supramolecular system based on NaphtGel, TTF‐PNIPAM, and CBPQT^4+^. AIBN=azobisisobutyronitrile.

The NaphtGel hydrogel, containing 3 mol % of naphthalene units, was prepared by the free‐radical copolymerization of a naphthalene‐functionalized acrylamide monomer and *N*,*N*‐dimethylacrylamide in the presence of *N*,*N′*‐methylenebisacrylamide (MBA; 0.8 mol %, as determined to be optimal for swelling; see Table S1 in the Supporting Information) as the cross‐linker (Figure 1 a; see the Supporting Information for further details). Owing to the low solubility of the naphthalene monomer, organogels were first prepared in *N*,*N*‐dimethylformamide, and were then washed with acetone, dried, and allowed to swell in water to give the NaphtGel hydrogel. As a control system, a hydrogel structure devoid of electron‐rich hydrophobic naphthalene units (NaphtGel0) was also prepared under the same synthetic conditions. The hydrogels were characterized by ^1^H NMR spectroscopy (see Figure S1 in the Supporting Information), which confirmed the presence of the naphthalene groups in a concentration of (3±0.6) mol % for NaphtGel, as estimated by integrating the signals of the naphthalene hydrogen atoms and the methylene fragments (‐C*H_2_*‐CH‐) of the polymer backbone (see the Supporting Information).

Next, we investigated the ability of the NaphtGel0 and NaphtGel hydrogels to swell in aqueous media and, more particularly, the impact of the presence of CBPQT^4+^ on their swelling ratio (*Q*; Figure 2 a,b; see the Supporting Information). As expected, owing to the presence of hydrophobic naphthalene units within the hydrogel structure, the NaphtGel displayed a slightly lower swelling ratio (*Q*=18±2) in water than NaphtGel0 (*Q*=20±2.5), which does not contain hydrophobic moieties (Figure 2 b). The addition of CBPQT^4+^ to NaphtGel0 did not significantly alter the swelling volume of this hydrogel (*Q*=25±4; Figure 2 b). In stark contrast, the addition of CBPQT^4+^ to NaphtGel led to a dramatic change in both the color (Figure 2 a) and the swelling ratio. In the presence of CBPQT^4+^, NaphtGel displayed the characteristic purple color of the naphthalene/CBPQT^4+^ donor–acceptor complex and a sharp increase in the swelling ratio from 18 to around 90. Besides these qualitative indications of host–guest complexation, ^1^H NMR (Figure 2 c) and UV/Vis spectroscopy (see Figure S7) confirmed the quantitative 1:1 host–guest complexation of the available naphthalene units with CBPQT^4+^.

**Figure 2:**
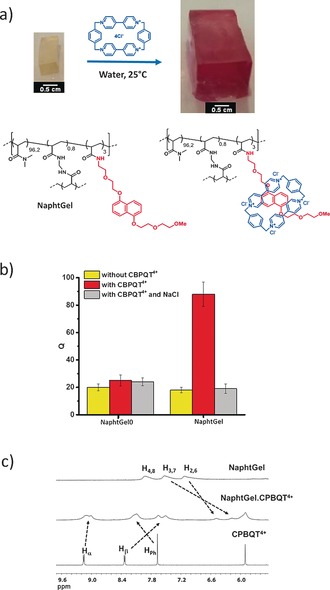
a) Photographs showing the swelling of NaphtGel after immersion in an aqueous solution of CBPQT^4+^ (3.7 mm) for 2 h and subsequent immersion in deionized water at 25 °C for 24 h. b) Swelling ratios (*Q*) of uncomplexed and complexed hydrogels after immersion for 24 h in water with and without NaCl (300 g L^−1^; 5.2 m). c) Partial ^1^H NMR spectra of NaphtGel (top), NaphtGel⋅CBPQT^4+^(middle), and CBPQT^4+^ (bottom) in D_2_O at 25 °C.

To obtain further insight into the mechanism of the CBPQT^4+^‐induced swelling of NaphtGel, we repeated the swelling experiment at different ionic strengths in the presence of [NaCl] (0–6.1 m) to examine the electrostatic interactions and osmotic pressure (Figure 2 b; see also Figure S6).^34^ Upon the addition of NaCl, the complexed gel kept its initial purple color, but a dramatic decrease in its swelling ratio was observed with salt concentrations well below 1 m, thereby clearly indicating that the swelling behavior of the gel is governed by the ionic contribution of the complexed CBPQT^4+^ host molecules and their chloride counterions.

Having demonstrated the ability of NaphtGel to swell by forming pseudorotaxane‐like architectures with CBPQT^4+^, we next turned our attention to the responsive and dynamic behavior of this complexed hydrogel. At first, the temperature responsiveness was evaluated by heating NaphtGel with CBPQT^4+^ from 5 °C up to 40 °C, which led to minor shrinkage of the gel and retention of the purple color, thus confirming that NaphtGel complexed with CBQT^4+^ is stable and is not thermoresponsive (see Figure S8). Next, the dynamic behavior of the complexes of CBPQT^4+^ with the naphthalene units in NaphtGel was studied by exposing a piece of complexed gel to a solution of TTF‐PNIPAM (*M*_n,NMR_=6400 g mol^−1^, *Đ*=1.1; see the Supporting Information for further details) at 8 °C, which is well below the cloud‐point temperatures (*T*_cp_) of both TTF‐PNIPAM (*T*_cp1_≈17.5 °C) and TTF‐PNIPAM complexed with CBPQT^4+^ (*T*_cp2_≈25 °C; see Figure S5). Owing to the strong binding affinity of CBPQT^4+^ towards TTF‐PNIPAM (association constant, *K*_a_=1.58±0.07×10^6^ m ^−1^; see Figure S4), CBPQT^4+^ was transferred from the hydrogel to the TTF‐PNIPAM‐containing solution, as clearly observed by the loss of the purple color of the hydrogel and the green coloration of the solution, the latter being indicative of the TTF–CBPQT^4+^ complex. These visual observations were confirmed by UV/Vis spectroscopy, which showed a clear shift of the absorption band from approximately 520 to 800 nm, indicative of the complexes of CBPQT^4+^ with naphthalene and TTF, respectively,^35^, ^36^, ^37^ upon the addition of a TTF‐PNIPAM solution to NaphtGel complexed with CBPQT^4+^ (Figure 3). Furthermore, exposure of noncomplexed NaphtGel to the solution of TTF‐PNIPAM⋅CBPQT^4+^ for 24 h did not lead to coloration of the hydrogel nor to a change in the swelling ratio, thus confirming that the CBPQT^4+^ host units remained complexed with TTF‐PNIPAM and also indicating that the TTF‐PNIPAM⋅CBPQT^4+^ complexes did not significantly diffuse into the hydrogel (see Figures S9 and S10).

**Figure 3:**
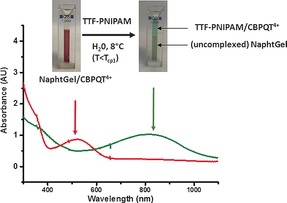
Photographs showing the color change of a sample consisting of an aqueous solution of NaphtGel/CBPQT^4+^ alone (left) and after exposure to TTF‐PNIPAM (0.5 mm) at 8 °C (*T*<*T*_CP1_) for 24 h (right), and UV/Vis spectra of a sample of NaphtGel/CBPQT^4+^ alone (red) and after exposure to TTF‐PNIPAM at 8 °C for 24 h (green).

Having demonstrated the CBPQT^4+^‐complexation‐induced swelling of NaphtGel, as well as the dynamic behavior of the supramolecular hydrogel complexation upon the addition of TTF‐PNIPAM, in a next step we investigated the heating‐induced swelling of NaphtGel in the presence of CBPQT^4+^ and TTF‐PNIPAM. This three‐component supramolecular system exploits the LCST‐induced dethreading process of TTF‐PNIPAM⋅CBPQT^4+^ complexes upon heating beyond its *T*_cp_ value, thus leading to the transfer of CBPQT^4+^ from TTF‐PNIPAM to NaphtGel and the accompanied swelling of the hydrogel, so‐called positive thermoresponsive behavior.

To probe the possibility of the temperature‐induced transfer of CBPQT^4+^ from TTF‐PNIPAM to NaphtGel, we heated a sample composed of the three‐component supramolecular system to 38 °C, which is above the *T*_cp_ value of the TTF‐PNIPAM⋅CBPQT^4+^ complex, and observed a sharp change in the color of the solution as well as the hydrogel (Figure 4 a). In particular, crossing of the *T*_cp_ value induced almost complete disappearance of the green color of the aqueous phase (which also became turbid as a result of the aggregation of TTF‐PNIPAM) and the appearance of the characteristic purple color of naphthalene‐based complexes within the hydrogel. These observations demonstrate that heating indeed induces release of the CBPQT^4+^ units by the LCST‐induced dethreading of the TTF‐PNIPAM⋅CBPQT^4+^ complexes, followed by transfer of CBPQT^4+^ to the NaphtGel, thus resulting in the formation of the corresponding naphthalene⋅CBPQT^4+^ complexes. After confirming that the TTF‐PNIPAM⋅CBPQT^4+^ complexes retained their stability at temperatures below the *T*_cp_ value by UV/Vis spectroscopy (see Figure S11), the kinetics of the temperature‐induced CBPQT^4+^ transfer was studied by monitoring the decrease in intensity of the absorption band of TTF‐PNIPAM⋅CBPQT^4+^ complexes in the aqueous phase of the three‐component supramolecular system at 8 °C (<*T*_cp_; *t*=0) and after heating to 38 °C (>*T*_cp_) for different time intervals (Figure 4 b; see Figure S12). From this experiment it is evident that the dethreading and transfer of the CBPQT^4+^ host to Naphtgel is a slow process that needs more than 10 h, most likely because of slow diffusion into the hydrogel (no agitation was applied).

**Figure 4:**
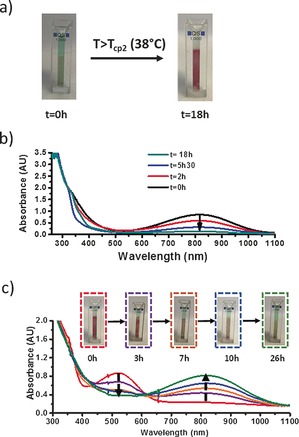
a) Photographs showing the color change of both a hydrogel piece and the supernatant of a sample composed of TTF‐PNIPAM⋅ CBPQT^4+^ (0.5 mm) and NaphtGel upon heating above *T*_cp2_ (38 °C) for 18 h (to induce complete transfer of CBPQT^4+^ from TTF‐PNIPAM to NaphtGel). b) UV/Vis spectra recorded at 8 °C on the supernatant of a sample composed of the three‐component supramolecular system after heating at 38 °C for 0, 2, 5.5, and 18 h. c) Photographs showing the color change and corresponding UV/Vis spectra with respect to time of a sample of TTF‐PNIPAM⋅CBPQT^4+^ and NaphtGel at 8 °C (<*T*_cp2_; *t*=0) upon heating at 38 °C (>*T*_cp2_) for 3, 7, 10, and 26 h.

Interestingly, the exchange of CBPQT^4+^ units between the TTF‐PNIPAM and the hydrogel was found to be fully reversible by cooling down the sample (Figure 4 c). Indeed, upon cooling of the three‐component system back to 8 °C, a gradual and opposite transfer of the host entities from the complexed NaphtGel to the free soluble TTF‐PNIPAM was observed through the change in color of both the solution and the hydrogel, which become yellowish and green, respectively. Furthermore, UV/Vis spectra recorded at 8 °C at different time intervals clearly showed the concomitant and progressive transition of the absorption band from naphthalene (520 nm) to TTF (800 nm) complexes. Again, the transfer process was slow and took more than 10 h. The reversibility of the dethreading–rethreading process was demonstrated during three successive heating/cooling cycles by monitoring the evolution of the absorption bands of both CBPQT^4+^‐based complexes (see Figure S13).

Once the reversible thermal control over the transfer of CBPQT^4+^ between TTF‐PNIPAM and NaphtGel had been established, we next investigated the extent of the macroscopic expansion and contraction of NaphtGel as controlled by this LCST‐induced host‐transfer process. Thus, a cube‐shaped hydrogel was immersed in an aqueous solution of TTF‐PNIPAM⋅CBPQT^4+^, and the expansion ratio *r*, defined as *r*=*L*(*T*)/*L*(8 °C), was determined at different temperatures (*T*=8 or 38 °C). Figure 5 a shows photographs of NaphtGel before and after successive exposure to a solution of TTF‐PNIPAM⋅CBPQT^4+^ at 8, 38, and 8 °C. As compared to the initial NaphtGel (*r*_0_=1), the complexed hydrogel resulting from the heat‐induced transfer of CBPQT^4+^ from TTF‐PNIPAM to NaphtGel displayed a significant increase in the *r* value (*r*_1_=1.6 *r*_0_), thus demonstrating that our supramolecular design indeed enables heating‐induced swelling of the hydrogel. This swelling process was found to be fully reversible upon cooling to 8 °C (*T*<*T*_cp_), which led to contraction of NaphtGel to its original size (*r*_2_≈1). Furthermore, the reproducibility of the swelling and contraction was confirmed over three heating/cooling cycles (Figure 5 b).

**Figure 5:**
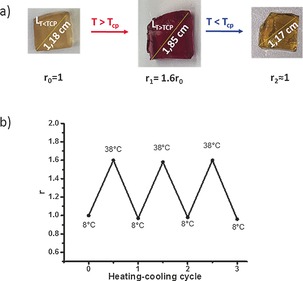
a) Photographs showing the macroscopic swelling of a cube‐shaped NaphtGel hydrogel upon successive immersion in a TTF‐PNIPAM⋅CBPQT^4+^ aqueous solution (0.5 mm) at 8, 38, and 8 °C (note that the pictures show the hydogels without the TTF‐PNIPAM solution). b) Plot of the expansion ratio (*r*) of the hydrogel over three successive heating–cooling cycles.

In conclusion, we have demonstrated that the supramolecular host CBPQT^4+^ effectively penetrated into a naphthalene‐functionalized hydrogel to form naphthalene–CBPQT^4+^ host–guest complexes, which resulted in a color change of the gel from pale yellow to purple and a sharp increase in the swelling ratio from 18 to 90. This swelling was found to be powered by the electrostatic contribution of the complexed CBPQT^4+^ units and its chloride counterions. By using the TTF‐PNIPAM⋅CBPQT^4+^ complex as a source of CBPQT^4+^ hosts, we gained temperature control over the swelling and contraction of the NaphtGel hydrogel. At temperatures below the *T*_cp_ value, the CBPQT^4+^ was complexed to TTF‐PNIPAM, and the uncomplexed NaphtGel was in the contracted state. Heating of this three‐component system led to the collapse of TTF‐PNIPAM above the *T*_cp_ value and release of the CBPQT^4+^ host, which diffused into the NaphtGel to form complexes with the naphthalene guests. As a result, NaphtGel underwent strong swelling when the temperature was increased. This fully reversible transition could be triggered on demand by varying the temperature below and above the polymer *T*_cp_ value. Importantly, the transition temperature may readily be tuned by varying the nature of the TTF‐functionalized thermoresponsive polymer. Moreover, the ability of TTF to undergo oxidation at low potentials may enable the use of electrical potential as input for the on‐demand (de)swelling of these and related gels.^38^ The introduction of supramolecular stimuli‐responsiveness into these hydrogel systems opens a wide variety of possibilities as components for advanced materials, nanotechnology, and biomedicine applications. Applications of this type are currently under development in our laboratories. Furthermore, our ongoing research efforts will focus on tuning of the transition temperature, acceleration of the CBPQT^4+^‐transfer process (which is still rather slow), and the combination of all components into a covalently linked single system so that it may be used in an open system without the loss of either the controlling polymer or the host.

## Supporting information

As a service to our authors and readers, this journal provides supporting information supplied by the authors. Such materials are peer reviewed and may be re‐organized for online delivery, but are not copy‐edited or typeset. Technical support issues arising from supporting information (other than missing files) should be addressed to the authors.

Supplementary
