# Supplementary material for: Recognition‐Mediated Hydrogel Swelling Controlled by Interaction with a Negative Thermoresponsive LCST Polymer
Source: Angew Chem Int Ed Engl. 2016 Oct 12;55(45):13974–8. doi: 10.1002/anie.201605630 (PMC5113761; doi:10.1002/anie.201605630)
Supplement: Supplementary file 1 — Supplementary [file ANIE-55-13974-s001.pdf]

Angewandte Chemie
Eine Zeitschrift der Gesellschaft Deutscher Chemiker

Supporting Information

Recognition-Mediated Hydrogel Swelling Controlled by Interaction with a Negative Thermoresponsive LCST Polymer

Khaled Belal, François Stoffelbach, Joël Lyskawa, Matthieu Fumagalli, Dominique Hourdet, Alba Marcellan, Lieselot De Smet, Victor R. de la Rosa, Graeme Cooke, Richard Hoogenboom,* and Patrice Woisel**

anie_201605630_sm_miscellaneous_information.pdf

Page contents

Experimental Section

1. Materials and instrumentation S3

2. Synthesis and characterization of NaphtGel and NaphtGel0 S4

3. Synthesis and characterization of TTF-PNIPAM S6

4. Determination of the association constant (K_a) and the LCST of TTF-PNIPAM/CBPQT^{4+} S8

5. Tcp of TTF-PNIPAM and TTF-PNIPAM/CBPQT^{4+} S8

6. Swelling and UV-Vis studies S9

-Effect of crosslinker on the swelling ratio of **NaphtGel** S9

- Effect of [NaCl] on the swelling ratio of **NaphtGel0**, **NaphtGel** and **NaphtGel/CBPQT^{4+}** S9
- UV-Vis binding studies between **NaphtGel** and **CBPQT^{4+}** S10
- Influence of the temperature on the swelling properties of **NaphtGel** and **NaphtGel/CBPQT^{4+}** S10
- Study of the dynamic behavior between **NaphtGel** and **TTF-PNIPAM/CBPQT^{4+}** at $T<T_{cp}$ and its influence on the swelling ratio of **NaphtGel** S11
- Influence of the temperature on **TTF-PNIPAM.CBPQT^{4+}** complexes S12
- Reversibility and reproducibility of the dethreading-rethreading process S13

7. References S14

Experimental Section

1. Materials and instrumentation

Materials:

All reagents were purchased from Sigma-Aldrich and used without further purification unless otherwise noted. *N,N*-Dimethylacrylamide (DMAC) was purified by vacuum distillation under reduced pressure before use. *N*-Isopropylacrylamide (NIPAM) was purified by recrystallizing twice from a mixture of toluene/hexane (60:40, v/v) and was dried under vacuum for 24h before use. The primary radical source used in all polymerizations was 2,2'-azobis(isobutyronitrile) (AIBN, > 98%, Fluka), which was recrystallized before RAFT polymerization.

Compounds NaphtAm,[1] TTF-CTA[2] and $CBPQT^{4+}$, $4Cl^{-}$ [3] were prepared according to literature procedures.

Instrumentation:

^{1}H NMR experiments were carried out on a Bruker Advance 400 spectrometer.

UV-Vis measurements were carried out on a Varian Cary 50 Scan UV/vis spectrophotometer equipped with a single cell Peltier temperature controller.

Size exclusion chromatography (SEC). The number-average molar masses (M_n), the weight-average molar masses (M_w), and the dispersities ($Đ$ = M_w/M_n) of the TTF-PNIPAM was determined by SEC. Measurements were performed in DMF (+ LiBr, 1 g L^{-1}) at 60 °C, at a flow rate of 0.8 mL min^{-1} and at a polymer concentration of 5 mg mL^{-1} after filtration through a 0.22 µm pore-size membrane. The chromatography was carried out on two PSS GRAM 1000 Å columns (8 × 300 mm; separation limits: 1 to 1000 kg mol^{-1}) and one PSS GRAM 30 Å (8 × 300 mm; separation limits: 0.1 to 10 kg mol^{-1}) coupled with three detectors (Viscotek, TDA 305): a differential refractive index (RI) detector, a viscosity detector and a light scattering (LS) detector (laser λ = 670 nm at 7° and 90°). The number-average molar masses, M_n^{PMMA}, and the dispersities ($Đ$ = M_w/ M_n), were calculated with a calibration curve based on narrow poly(methyl methacrylate) (PMMA) standards (from Polymer Standard Services), using only the RI detector. The absolute number-average molar masses, M_n^{LS}, were calculated using the RALS/LALS and RI signals.

Isothermal titration calorimetry (ITC) experiments were performed using a nano-ITC titration calorimeter from TA Instruments with a standard sample cell volume of 1 mL at 8 °C. A 250 μL injection syringe was used with stirring at 400 rpm. TTF-PNIPAM (0.22 mM) was dissolved in deionized water and the solutions were degassed gently under vacuum before use. Each titration comprised 25 × 10 μL injections of $CBPQT^{4+}$ (1.57 mM) into guest solution. Control experiments with identical injections of $CBPQT^{4+}$ into deionized water alone were used to correct titration data. ITC data collected during the analysis were fitted to an independent sites model where one independent site binds one ligand. The fit and the determination of thermodynamic parameters (K_a, n, ΔH, ΔS, ΔG) were achieved by using the TA Instrument ITC analysis software (NanoAnalyze).

Turbidity measurements were performed on a Cary 50 UV-Visible spectrophotometer with Peltier temperature control at a wavelength of 540 nm. The samples were first cooled to a suitable temperature to fully dissolve the polymer (0,5 mM) in deionized water, after which the sample was placed in the instrument and cooled to 5°C. In the case of supramolecular host-guest complexes, 1 equivalent of $CBPQT^{4+}$ was added to polymer solution. The transmittance was measured during at least two controlled cooling/heating cycles with a cooling/heating rate of 1 °C min^{-1} from 5°C to 40°C with hold steps of 5 min at the extreme temperatures while stirring. T_{CP} is given as the temperature when the transmittance drops to 50% during the second heating ramp.

2. Synthesis and characterization of NaphtGel and NaphtGel0

Synthesis of NaphtGel

N,N-Dimethylacrylamide (1 g, 10 mmol), AIBN (8.6 mg, 0.052 mmol), *N,N'*-methylenebisacrylamide (0.0128 g , 0.083 mmol) and NaphtAm[1] (0.122 g, 0.3 mmol) were stirred in DMF (4.5 g) in a sealed flask. The mixture was deoxygenated by nitrogen bubbling for 20 min in an ice/water and the solution was transferred between two glass plates (7 x 3 cm) placed into a sealed flask under nitrogen. The reactor was heated for 24 h at 70 °C in an oven. Then, **NaphtGel** was removed from the glass plates at room temperature and washed with acetone and dried successively at room temperature for 24 h and at 60 °C for 12 h. The dried gel was put in water and left under stirring for 5 days to swell at equilibrium. **NaphtGel0** was prepared using the same procedure without NaphtAm.

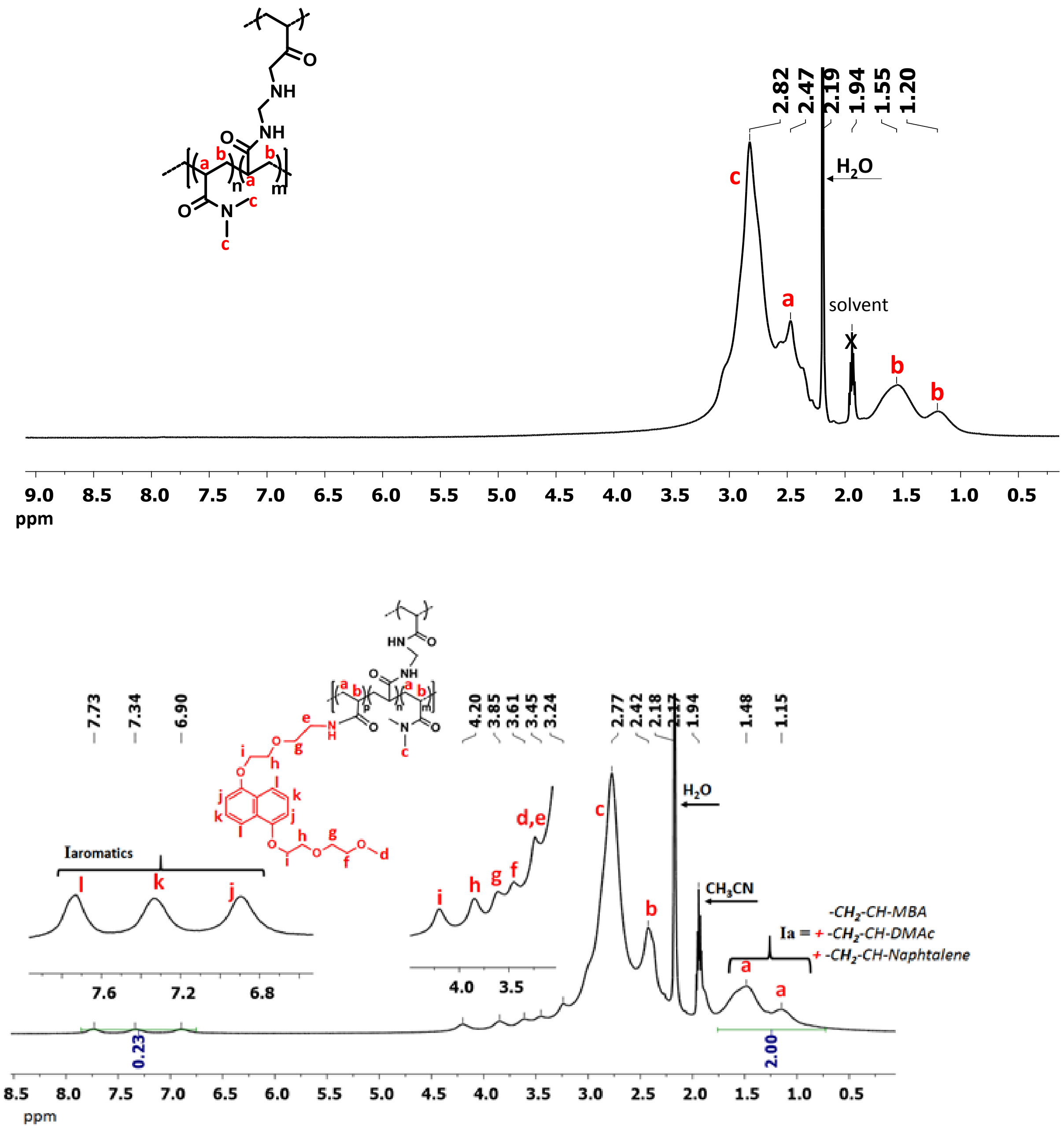

Figure S1: ^{1}H NMR spectra of **NaphtGel0** (top) and **NaphtGel** (bottom) recorded in CD_3CN at 25°C.

The molar percentage of NaphtAm units (mol% NaphtAm) in the copolymer was calculated with the following equation:

Equation 1. $$mol\%\ of\ NaphtAm = \frac{\text{Iaromatics}}{3*\text{Ia}} * 100$$

The mol% of NaphtAm for **NaphtGel** was estimated as 3 % (± 0.6).

3 Synthesis and characterization of TTF-PNIPAM

RAFT-mediated homopolymerization of NIPAM.

A mixture of NIPAM (4 g, 35 mmol), DMF (4 g), AIBN (14.5 mg, 0.09 mmol) and TTF-CTA[2] (200 mg, 0.44 mmol) was deoxygenated by bubbling argon for 30 min in ice/water bath and was then immersed in an oil bath at 70 °C. After 10 h, the reaction was stopped, and a sample was withdrawn to measure the conversion by ^{1}H NMR spectroscopy by comparing the integrated areas of characteristic signals of monomer and polymer. The polymer was isolated by precipitation into diethyl ether. After complete drying, the polymer was characterized by ^{1}H NMR spectroscopy in CD_3CN and by SEC in DMF (+ LiBr). $M_{n\ NMR}$ = 6420 g mol^{-1}. $M_n^{LS}{}_{SEC}$ = 6500 g mol^{-1} and $Đ$ = 1.1. (see Figure S2 and S3).

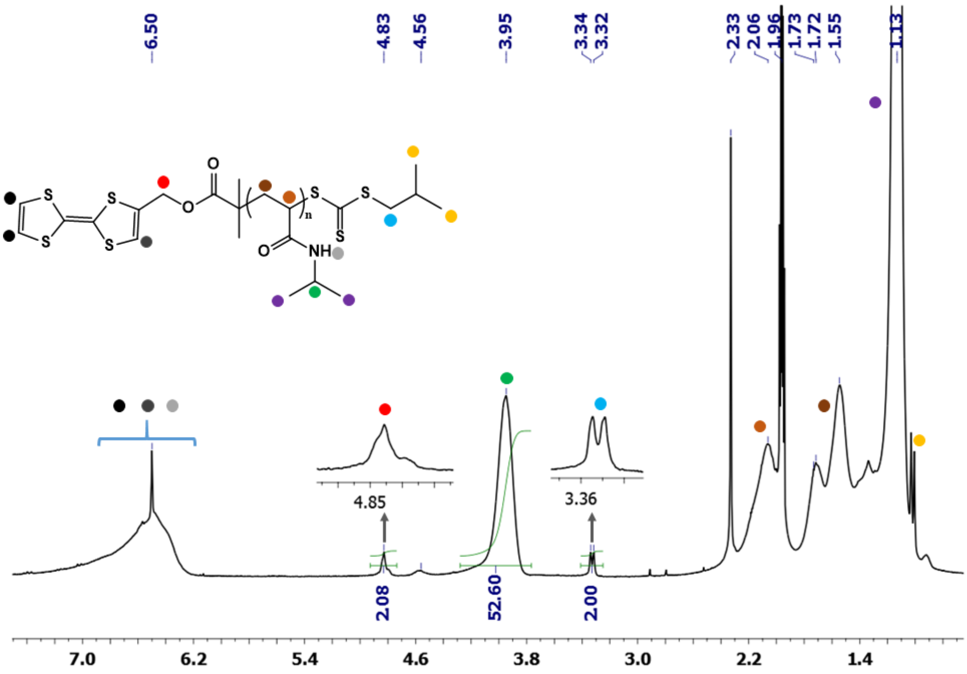

Figure S2: ^{1}H NMR spectrum of the **TTF-PNIPAM** recorded in CD_3CN at 25°C.

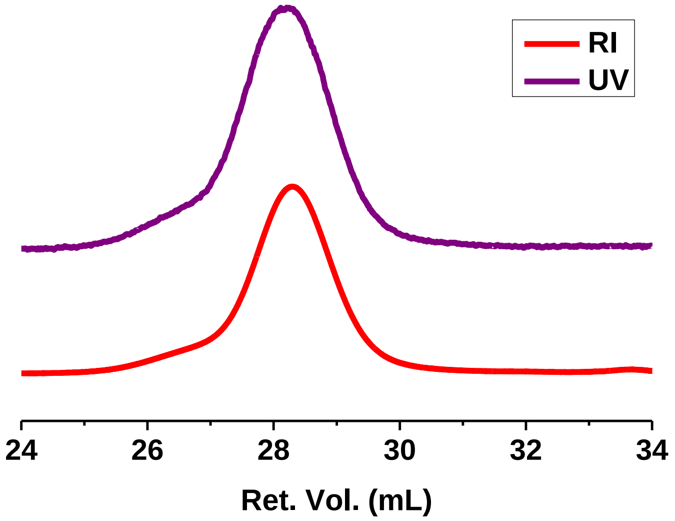

Figure S3: SEC chromatograms obtained with an UV detector at 254 nm (in purple) and a refractive index detector (in red) of **TTF-PNIPAM** after purification.

4. Determination of the association constant (K_a)

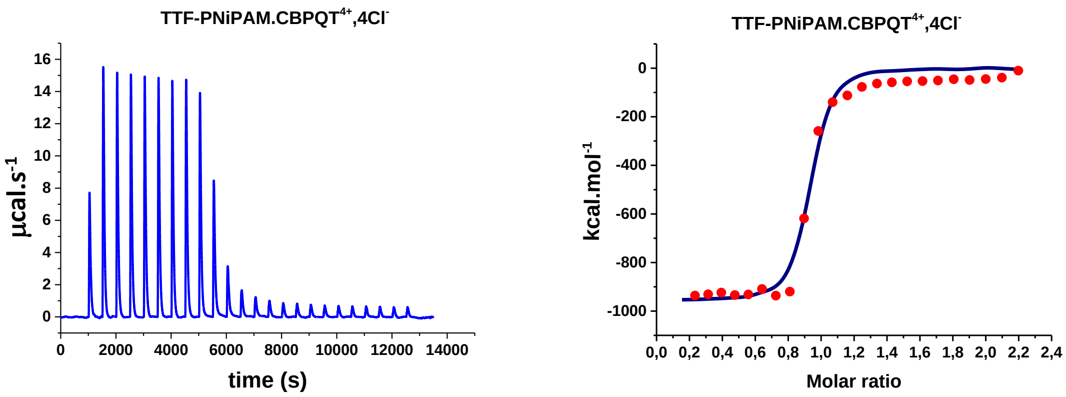

Fig. S4: Isothermal titration calorimetry data for the addition of aliquots of $CBPQT^{4+}$ (1.57 mM) to **TTF-PNIPAM** (0.22 mM). Recorded in H_2O at 8 °C (K_a = 1.58 ± 0.07 x10^6 M^{-1}, N = 0.91(± 0.01), ΔH= - 60.7(±2.3) $kJ.mol^{-1}$, ΔS= - 0.01(±0.003) $kJ.mol^{-1}$, ΔG= - 33.4 (±0.8) $kJ.mol^{-1}$).

5. Tcp of TTF-PNIPAM and TTF-PNIPAM/$CBPQT^{4+}$

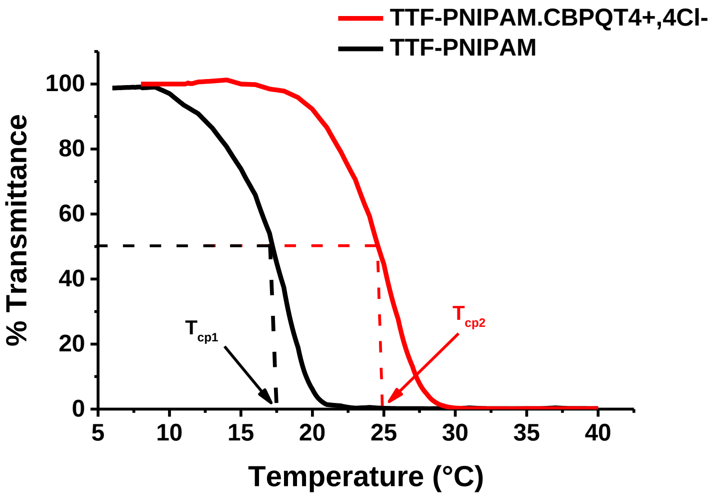

Figure S5: Transmittance (measured at 540 nm) as a function of temperature for **TTF-PNIPAM** (0.05 mM) (in black) and **TTF-PNIPAM/$CBPQT^{4+}$** (1eq.) (in red) in H_2O.

6. Swelling and UV-Vis studies

Hydrogel samples were swelled in deionized water at 25°C. The swelling ratios of the samples were determined gravimetrically at different times to follow the swelling kinetics using the following equation:

Equation 2. $$\text{Swelling ratio} = Q = \frac{m_S - m_D}{m_D}$$

with m_S, the weight of the swelled hydrogel and m_D, the weight of the hydrogel after drying to a constant weight at 60°C (the values are an average of three measurements). The equilibrium-swelling ratio was reached after 24h.

The required amount of $CBPQT^{4+}$, $4Cl^-$ to complex all naphthalene units within **NaphtGel** was calculated on the basis of the mol% of NaphtAm calculated by ^{1}H NMR spectroscopy.

Effect of crosslinker on the swelling ratio of NaphtGel

The amount of crosslinker in **NaphtGel** was optimized in order to obtain hydrogels with maximum swelling ratio. The results obtained by variation of the amount of MBA crosslinker are summarized in Table S1 revealing that 0.8 mol% crosslinker is optimal for this work as it is just sufficient for crosslinking while facilitating large chain motion and mobility as required for swelling.

Table S1. Effect of crosslinker on swelling ratio (Q) of **NaphtGel** hydrogels containing 3 mol% naphthalene functionalized monomer in absence and presence of $CBPQT^{4+}$.

mol% MBA crosslinker	Swelling without $CBPQT^{4+}$	Swelling with $CBPQT^{4+}$
< 0.8	No gel	No gel
0.8	18	90
2	14	25
4	6	9

Effect of [NaCl] on the swelling ratio of NaphtGel0, NaphtGel and NaphtGel/CBPQT^{4+}

After having reached their equilibrium swellings in deionized water, hydrogels pieces were immersed in different [NaCl] (from 0 to 357 g/L; 0 to 6.1 M) for 3 days and their new swelling ratios were estimated.

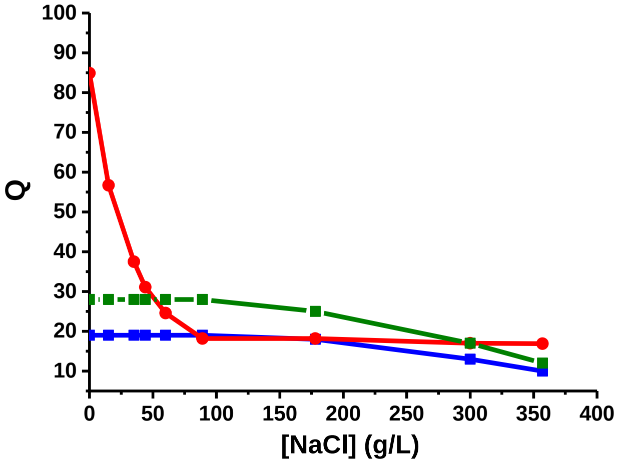

Figure S6: Evolution of the swelling ratio of **NaphtGel0** (in green), **NaphtGel** (in blue) and **NaphtGel/CBPQT^{4+}** (in red) with the [NaCl] at 25°C.

UV-Vis binding studies between NaphtGel and CBPQT^{4+}

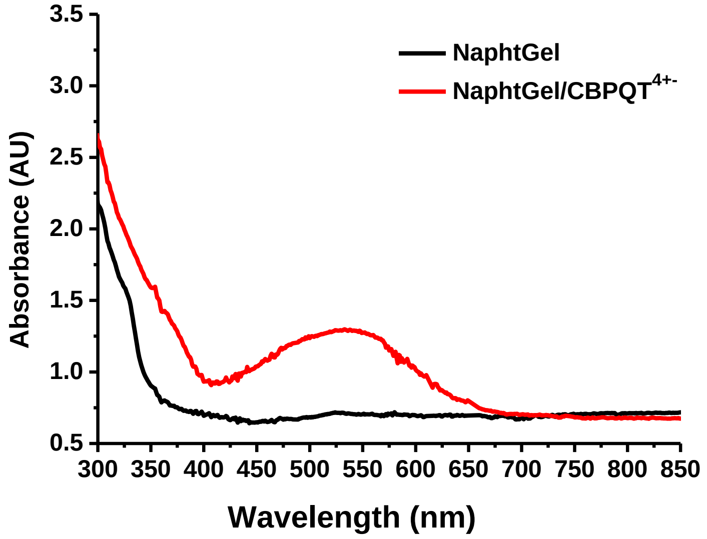

Figure S7: UV-Vis spectra of **NaphtGel** (in black) and **NaphtGel/CBPQT^{4+}** (in red) recorded in water at 25°C.

Influence of the temperature on the swelling properties of NaphtGel and NaphtGel/CBPQT^{4+}

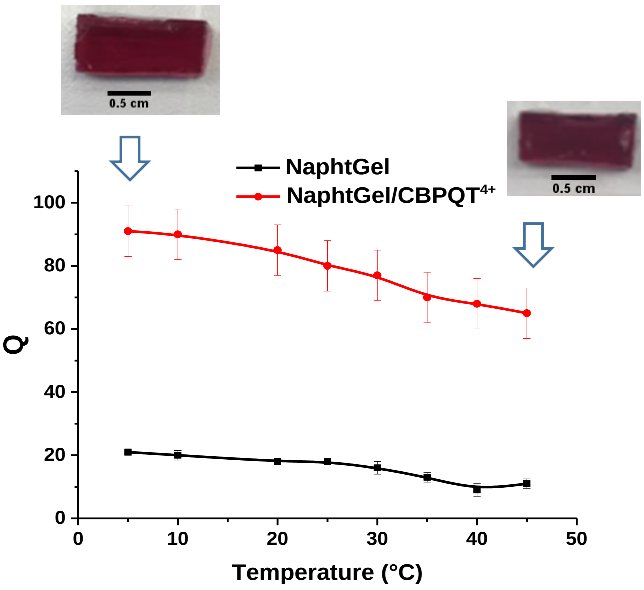

Figure S8: Photographs of **NaphtGel/CBPQT^{4+}** after being immersed in deionized water at 5°C (left) and 45°C (right) for 24h. Evolution of the swelling ratio of **NaphtGel** (in black) and **NaphtGel/CBPQT^{4+}** (in red) at different temperatures.

Study of the dynamic behavior between NaphtGel and TTF-PNIPAM/CBPQT^{4+} at T<T$_{cp2}$ and its influence on the swelling ratio of NaphtGel

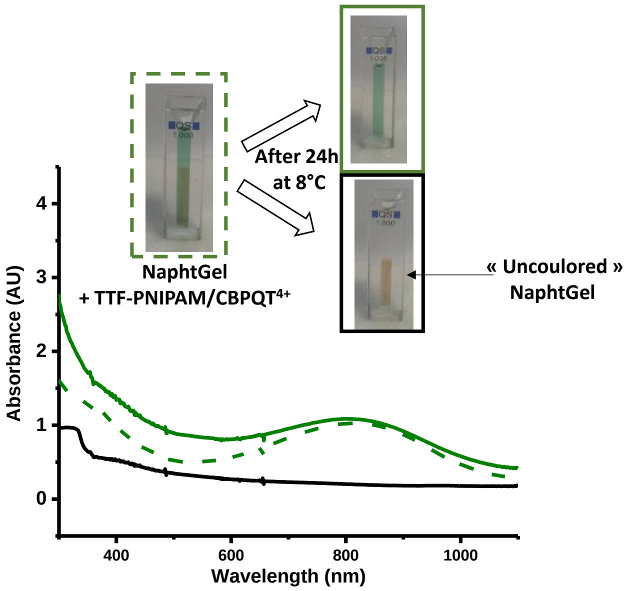

Figure S9: Photographs and UV-Vis spectra of an aqueous mixture of **NaphtGel** and **TTF-PNIPAM/ CBPQT^{4+}** (left**)** at 8°C and the resulting supernatant (top right) and the gel (bottom right) after 24h at 8°C.

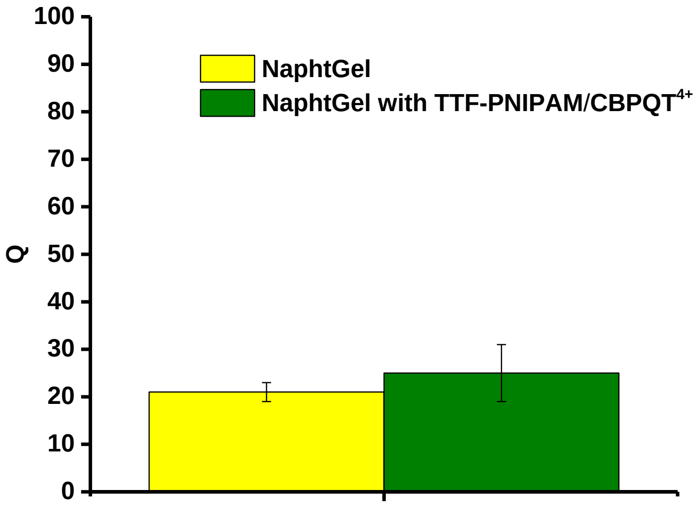

Figure S10. Swelling ratios of **NaphtGel** alone (in yellow) and after being exposed to a solution of **TTF-PNIPAM/CBPQT^{4+}** (in green) at 8°C for 24h.

Influence of the temperature on TTF-PNIPAM.CBPQT^{4+} complexes

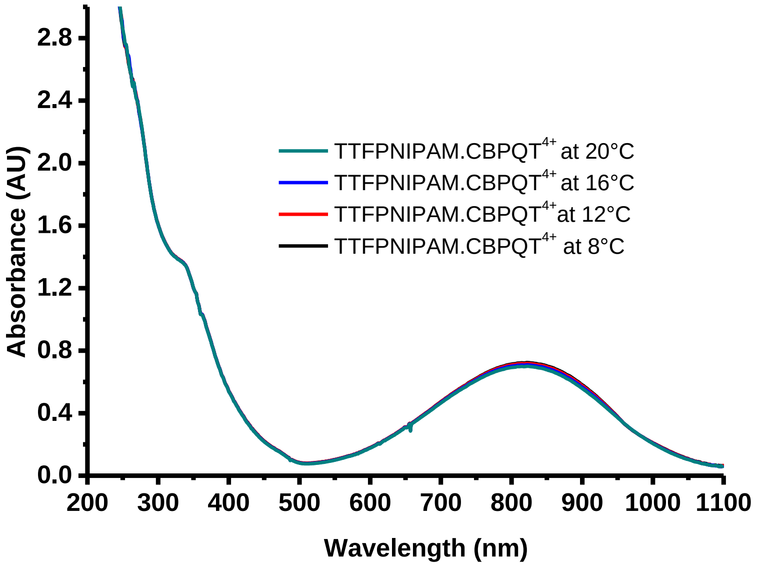

Figure S11: UV-Vis spectra of **TTF-PNIPAM/CBPQT^{4+}** at different temperatures (below the T_{cp2}).

Kinetic of the transfer of CBPQT^{4+} units from TTF-PNIPAM/CBPQT^{4+}complexes to NaphtGel at T>T$_{cp2}$

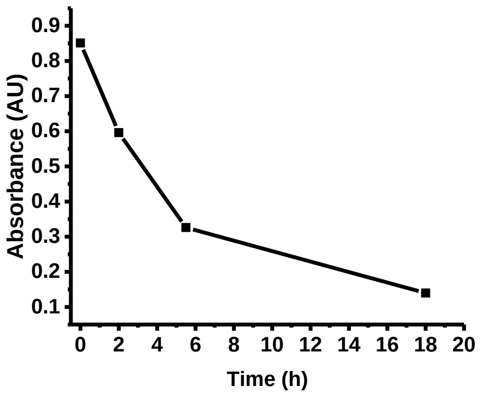

Figure S12: Evolution of the intensity of the absorption band centered at 800 nm *vs* time of the supernatant of a sample composed of the three-component supramolecular system after having been heated at 38 °C for 0 , 2, 5.5 and 18 hours.

Reversibility and reproducibility of the dethreading-rethreading process

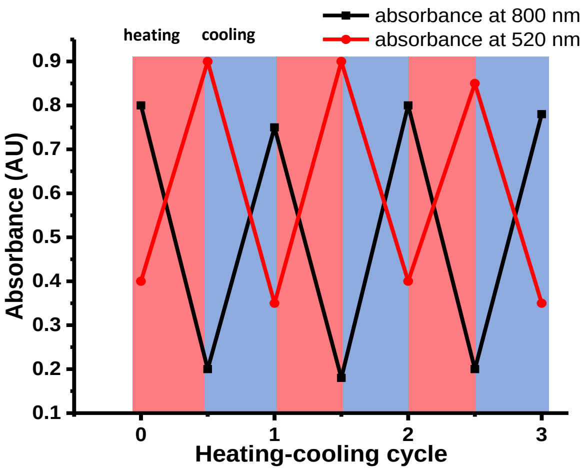

Figure S13: Plot of the relative absorbance intensities at 500 nm and 800 nm during three successive heating-cooling cycles.

7. References

1 L. Sambe, V. R. Delarosa, K. Belal, F. Stoffelbach, J. Lyskawa, F. Delattre, M. Bria, G. Cooke, R. Hoogenboom and P. Woisel *Angew. Chem., Int. Ed. Engl.*, 2014, **53**, 5044–5048.

2 J. Bigot, B. Charleux, G. Cooke, F. Delattre, D. Fournier, J. Lyskawa, F. Stoffelbach, and P. Woisel *Macromolecules* 2010, **43**, 82-90.

3 B. Odell, M. V Reddington, A. M. Z. Slawin, N. Spencer, J. F. Stoddart and D. J. Williams *Angew. Chem., Int. Ed. Engl.*, 1988, **100**, 1547–1550.
